# Outcomes of Endoscopic Sleeve Gastroplasty: A Systematic Review

**DOI:** 10.3390/medicina61101821

**Published:** 2025-10-11

**Authors:** Vanessa Pamela Salolin Vargas, Omar Thaher, Moustafa Elshafei, Sjaak Pouwels, Carolina Pape-Köhler

**Affiliations:** 1 Facultad de Medicina, Universidad Westhill, Mexico City 05610, Mexico; vanesalolin@gmail.com; 2 Department of Surgery, Knappschaft Kliniken University Hospital Bochum, Ruhr-University Bochum, 44892 Bochum, Germany; 3 Department of General, Visceral and Transplant Surgery, Muenster University Hospital, Muenster University, 48149 Muenster, Germany; 4 Department of General, Visceral and Tumor Surgery, St. Elisabethen Hospital Frankfurt am Main, 60487 Hessen, Germany; 5 Department of Surgery, Bielefeld University—Campus Detmold, Klinikum Lippe, 32756 Detmold, Germany; 6 Department of Intensive Care Medicine, Elisabeth-Tweesteden Hospital, 5022 GC Tilburg, The Netherlands; 7 Bariatric Clinic, Klinikum Bielefeld—Rosenhöhe, 33647 Bielefeld, Germany; 8 Department of General and Visceral Surgery, Klinikum Bielefeld—Mitte, Medical School and University Medical Center OWL, Bielefeld University, 33615 Bielefeld, Germany

**Keywords:** endoscopic bariatric therapy, obesity, intragastric balloons, endoscopic sleeve gastroplasty

## Abstract

*Background and Objectives*: Endoscopic sleeve gastroplasty (ESG) is a minimally invasive endoscopic procedure that has demonstrated both safety and effectiveness in the treatment of obesity. By reducing the stomach’s volume without the need for surgical incisions, ESG promotes weight loss and can improve obesity-related comorbidities. However, patient responses to ESG can vary significantly. *Materials and Methods*: A comprehensive search was performed on PubMed, Embase, and Cochrane for studies with endoscopic sleeve gastroplasty; the main outcomes of interest are BMI, weight loss, and postinterventional complications. The search strategy employed a combination of keywords and Medical Subject Heading (MeSH) terms, including “endoscopic sleeve gastroplasty,” “endoscopy,” and “overweight”. To ensure the thoroughness of the review, additional manual searches of key journals and the reference lists of identified studies were performed. Grey literature, such as dissertations and conference abstracts, meta-analysis, and systematic reviews, was excluded to maintain a focus on peer-reviewed evidence. Duplicate records were identified and removed using Rayyan software to streamline the screening process. The I2 test was employed for heterogeneity assessment, while the risk of bias was evaluated utilizing ROBINS-I. *Results*: Our literature search resulted in the inclusion of 38 studies. Endoscopic sleeve gastroplasty for weight loss is important since it is more effective than pharmacological treatments and lifestyle changes and presents lower adverse event rates compared to bariatric surgery. Long-term weight loss outcomes varied, with total body weight loss ranging from 16% to 20.9% over a period from 2 to 5 years, while excess weight loss ranged from 13% to 79%. Revisional procedures showed higher failure rates, with up to 34.3% of patients experiencing insufficient weight loss. Most interventions led to clinically significant and sustained weight loss, though variability in outcomes highlights the need for further research to optimize long-term weight management strategies. *Conclusions*: Endoscopic sleeve gastroplasty (ESG) emerges as a promising minimally invasive option for weight loss, offering significant improvements in both weight reduction and obesity-related comorbidities, such as diabetes, hypertension, and dyslipidemia.

## 1. Introduction

Obesity, defined as having a body mass index (BMI) of 30 kg/m^2^ or higher, is recognized as a global public health challenge by the World Health Organization (WHO) [1]. In 2014, 13% of adults aged 18 and older were classified as having obesity, while an additional 39% were categorized as being overweight (BMI > 25). Despite being theoretically preventable, approximately 650 million individuals worldwide were living with obesity at that time [2]. By 2030, nearly one in two adults are predicted to have obesity [1,2].

The development of obesity is influenced by a combination of genetic predisposition, prolonged unhealthy dietary patterns, and a lack of physical activity [3]. This complex condition is linked to various health issues, including type 2 diabetes, high blood pressure, fatty liver disease, and other cardiovascular disorders [3].

The primary approach to treating obesity involves implementing dietary plans and adopting lifestyle changes to encourage increased physical activity. Achieving meaningful results requires consistent patient adherence, as the process is often lengthy and demanding [4,5,6]. However, these measures alone are often inadequate for achieving significant weight loss, necessitating additional interventions.

Medications designed to enhance feelings of fullness can complement dietary strategies, but their effectiveness is generally limited. Furthermore, the potential for adverse side effects often restricts their long-term use and there is the possibility of regaining weight after stopping the medications [3,4,5,6].

Endoscopic sleeve gastroplasty (ESG) is a minimally invasive transoral procedure that replicates the restrictive effects of bariatric surgery [6]. ESG has demonstrated effectiveness in achieving weight loss and improving obesity-related comorbidities while maintaining a favorable safety profile [6]. ESG may offer a viable alternative for elderly patients who are not candidates for traditional bariatric surgery. In addition, a safe procedure was defined for children and adolescents [6].

Bariatric surgery has proven to induce a long-term and durable effect in terms of weight loss, which can be up to 25–30% of initial weight. These results are shown to be stable over the long term [7].

The goal of this systematic review is to provide a comprehensive understanding of ESG as a non-surgical weight loss procedure. It seeks to explore the advantages, potential risks, eligibility factors, and expected outcomes of ESG, while examining how it stands apart from other weight loss interventions.

## 2. Materials and Methods

We conducted a multi-database literature search using a comprehensive search strategy based on the PICO (Patient; Intervention; Comparison; and Outcome) acronym. The patient population of interest was all patients with obesity and patients undergoing endoscopic surgery techniques. The intervention studied was endoscopic sleeve gastroplasty. The outcome measurements studied were weight loss (and related changes in anthropometric variables) and comorbidity resolution.

PubMed, Medline, and the Cochrane Library were searched from the earliest date of each database up to 6 January 2025. We used a search string containing the following keywords: (((overweight) OR obesity)) AND (((endoscopic) OR endoscope) OR endoscopic sleeve gastroplasty). The search string was modified for each database when necessary.

Initial screening and selecting studies based on title and abstract was completed by authors VPSV and SP. After the primary selection round, both authors individually reviewed the full text of each article and determined suitability for inclusion in the systematic review. Cross-references were studied to look for further eligible studies. Disagreements were solved by discussion with co-authors until consensus was reached.

### 2.1. Eligibility Criteria

The population included adult patients undergoing endoscopic sleeve gastroplasty, while studies involving pediatric populations or animals were excluded. Clinical outcomes assessed included postoperative complications (both infectious and noninfectious), length of hospital stay, wound healing, and original studies. Studies were excluded if they were abstracts, editorials, meta-analyses, poster presentations, narrative reviews, or preprints.

### 2.2. Risk of Bias Assessment

We evaluated the quality assessment in non-randomized studies with the Risk of Bias in Non-Randomized Studies of Interventions tool (ROBINS-I). Two independent authors completed the risk of bias assessment (authors VPSV and SP). Disagreements were resolved through a consensus after discussing reasons for the discrepancy.

### 2.3. Endoscopic Sleeve Gastroplasty Definition and Technique

Over the past decade, ESG has emerged as a method for managing obesity, most notably among individuals with class I and II obesity (BMI 30–39.9 kg/m^2^), aligning with standard classifications of mild to moderate obesity. In recent years, it has gained prominence as an effective, minimally invasive, and cost-effective alternative to bariatric surgery for weight loss [8].

ESG involves placing full-thickness sutures along the stomach’s greater curvature, reshaping it into a sleeve-like structure, and reducing its volume by approximately 80% [9]. ESG has demonstrated notable effectiveness in individuals with class II obesity, leading to an average total body and weight reduction of around 16% within a year [9].

In July 2022, the United States Food and Drug Administration (FDA) approved the Apollo ESG™ system (formerly the OverStitch device by Apollo Endosurgery, Austin, TX, USA) through De Novo Market Authorization for managing obesity in individuals with a BMI between 30 kg/m^2^ and 50 kg/m^2^. However, due to the relatively recent development of endoscopic bariatric procedures and earlier guidelines favoring their use in patients with lower obesity categories, research on the effectiveness of ESG in class III obesity remains scarce [8,10]. Several studies highlight that ESG, combined with a structured lifestyle modification program (diet and exercise counseling) can lead to significant weight loss in patients with obesity. Especially in high-risk patients, this can be beneficial and the intervention in general has a less invasive character [8,10,11,12,13,14].

## 3. Results

The initial literature search produced 751 results, including three duplicates. After screening titles and abstracts, 38 studies were included.

**Figure 1:**
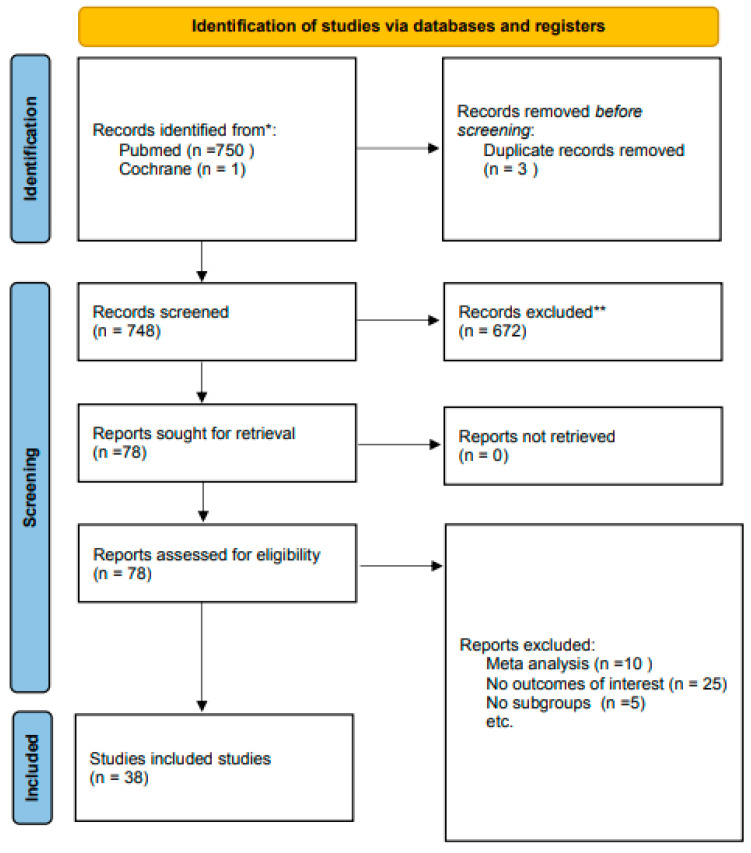
PRISMA flow diagram.

**Figure 2:**
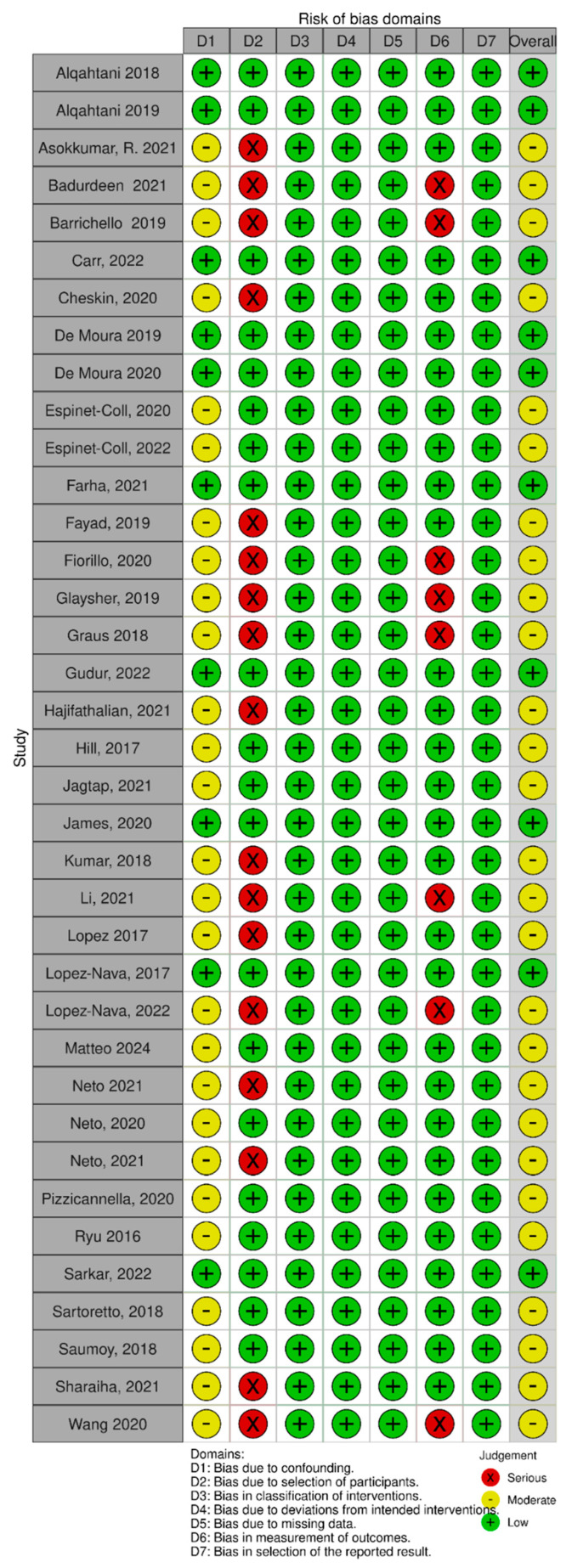
Assessment of methodological quality using the Risk Of Bias in Non-Randomized Studies of Interventions scale (ROBINS-I) for non-randomized trials.

Thirty-eight studies [4,5,8,15,16,17,18,19,20,21,22,23,24,25,26,27,28,29,30,31,32,33,34,35,36,37,38,39,40,41,42,43,44,45,46,47,48,49,50] were included in this systematic review. Figure 1 summarizes the search results, according to Preferred Reporting Items for Systematic Reviews and Meta-Analyses (PRISMA) guidelines [51]. The methodological quality of the included studies ranges from low to moderated, indicated by the Risk Of Bias in Non-Randomized Studies of Interventions scale (ROBINS-I) for non-randomized trials (Figure 2). A Cohen’s kappa of 0.75 reflected a good agreement between authors (between authors V.S. and S.P.). Table 1 gives an overview of the results of the included studies. Due to significant heterogeneity among the included studies, a meta-analysis was not performed.

Of the 38 studies included, one was a retrospective cohort study [5], two were retrospective reviews [4,32], two were multicenter retrospective studies [16,20], one was a multicenter prospective study [4], one was a prospective observational study [22], one was a prospective cohort study [21], one was a retrospective single-center study [17], and one was a multicenter randomized trial [11]. Six were case series [8,23,24,25,26,27], one was a cohort study [28], thirteen were prospective studies [30,31,33,36,37,38,40,41,43,44,45,46,49], and eight were retrospective studies [24,34,35,39,42,47,48,50]. All references were organized and managed using Mendeley to ensure accurate citation and straightforward access to source materials.

The majority of the included studies were retrospective in design, focusing on analyzing outcomes of bariatric procedures and their associated effects.

### Weight Loss and Comorbidity Resolution

Endoscopic sleeve gastroplasty (ESG) has emerged as a promising minimally invasive intervention for the management of obesity, demonstrating consistent effectiveness in inducing significant weight loss and improving metabolic parameters. Across multiple studies, ESG has led to meaningful reductions in total body weight, body mass index (BMI), and obesity-related comorbidities.

Weight loss outcomes vary depending on study design and patient population, but the overall trend supports ESG’s efficacy. De Moura et al. [4], in a multicenter prospective study of 233 patients, reported an average total body weight loss (TBWL) of 19.7%, a result consistent with other large-scale findings. Matteo et al. [5], analyzing a retrospective cohort of 315 patients, found TBWL ranging from 17% to 20% at 2 years, with a sustained loss of 16% at 5 years, highlighting ESG’s long-term durability.

In addition, ESG has been associated with metabolic improvements, including better glycemic control, lipid profile normalization, and reduced blood pressure in patients with obesity-related conditions such as type 2 diabetes and hypertension. Though specific metabolic markers were not uniformly reported across all studies, weight loss of ≥10% is generally correlated with these benefits, and many ESG trials exceeded this threshold.

Beyond weight loss, ESG has been associated with meaningful improvements in obesity-related comorbidities. Adult populations have reported remission or significant improvement in conditions such as type 2 diabetes mellitus, hypertension, dyslipidemia, and obstructive sleep apnea following ESG [21]. However, even moderate reductions of 10–15% in total body weight have been shown to produce clinically relevant improvements in insulin sensitivity, blood pressure regulation, and lipid profiles [21].

From a safety perspective, ESG is well tolerated, with a lower risk profile compared to surgical bariatric interventions. Most studies reported mild-to-moderate adverse events, such as nausea and abdominal discomfort, with very low rates of serious complications. This favorable safety profile makes ESG an attractive alternative for patients who are either ineligible for or unwilling to undergo surgery.

In summary, ESG consistently results in substantial weight loss—typically from 13% to 20% TBWL at 12 months—with additional benefits including BMI reduction, improved obesity-related comorbidities, and a low complication rate. These outcomes reinforce ESG’s role in modern obesity management and support its consideration as part of a personalized treatment strategy.

## 4. Discussion

The studies primarily focused on the outcomes of ESG for obesity, with additional studies covering weight regain after sleeve gastrectomy, comparisons between ESG and laparoscopic sleeve gastrectomy, and ESG’s efficacy and safety. The clinical utility ESG has been increasing over the last few years due to the procedure’s safety and efficacy for the treatment of patients with obesity. This was illustrated by several studies in the literature [52]. However, it should be taken into account that, in the current literature, there is a lack of controlled studies directly comparing ESG with established bariatric procedures, such as laparoscopic sleeve gastrectomy or gastric bypass.

Obesity is a chronic and multifactorial condition linked to numerous comorbidities, with its prevalence steadily increasing among the elderly population [53]. In geriatric individuals, obesity can lead to accelerated declines in physical function, reduced quality of life, heightened risk of institutionalization, increased mortality rates, and a significant financial burden on the healthcare system [54]. For many years, voluntary weight loss in the elderly has been considered undesirable, primarily due to concerns about the potential loss of muscle mass and the associated risk of deteriorating functional status. This apprehension stems from the fact that, as individuals age, maintaining muscle strength and functional mobility becomes increasingly important for maintaining independence and quality of life. It needs to be stated that ESG has also been used successfully in children and adolescents illustrating similar weight loss profiles and remission of obesity-related comorbidities [55]. Roughly, it can be stated the weight loss induced by ESG is the result of reducing the stomach’s capacity by approximately 80% and, therefore, altering the stomachs physiology. One of the most important effects is delaying gastric emptying [56].

ESG is a non-surgical minimally invasive procedure that involves the placement of full-thickness sutures in the stomach to create a smaller, sleeve-like shape. ESG has gained popularity as a less invasive alternative for patients seeking long-term weight loss solutions, offering benefits such as a shorter recovery time and lower risk of complications compared to conventional bariatric surgery [5,7]. This procedure effectively reduces the stomach’s capacity, limiting food intake and promoting weight loss. ESG has been shown to be highly effective in inducing weight loss both in the short and medium term, with significant improvements in obesity-related comorbidities such as diabetes, hypertension, and hyperlipidemia [8] Additionally, ESG offers the advantage of being less invasive than traditional bariatric surgeries, with a lower risk of complications, making it a promising option for patients seeking an alternative to more invasive weight loss procedures [51].

In terms of weight loss, ESG typically leads to a total body weight loss (TWL) of approximately 20–25% and excess weight loss (EWL) ranging from 40% to 60% within the first 12 to 24 months [17]. These outcomes are similar to those achieved with more invasive bariatric procedures, such as laparoscopic sleeve gastrectomy, but with the added benefit of being minimally invasive [16,18]. ESG’s restrictive effects on the stomach, by reducing its capacity, limit food intake and slow gastric emptying, leading to increased feelings of fullness and reduced appetite, which contributes to weight loss [19].

In addition to weight reduction, ESG has shown considerable success in improving obesity-related comorbidities. One of the most notable benefits is the improvement in type 2 diabetes, with several studies reporting remission or significant reduction in blood sugar levels post procedure [4]. Hypertension and dyslipidemia have also been shown to improve, contributing to better cardiovascular health and a reduced risk of complications associated with these conditions. In fact, some patients have been able to stop or reduce their medications for diabetes, high blood pressure, and cholesterol [5,6].

However, ESG provides a promising alternative to traditional bariatric surgery, particularly for patients who are looking for a less invasive reversible option [19]. The weight loss and health benefits associated with ESG make it a valuable tool in the management of obesity and its related conditions, offering patients significant improvements in both physical health and quality of life. In addition, the method may be appropriate as an alternative less invasive procedure for patients who, due to their limited health condition, are at a higher risk of negative side effects from anesthesia and surgery and are not candidates for bariatric surgery. Another key advantage of ESG is that the procedure can easily be repeated if weight regain occurs [57,58].

## 5. Limitations

Despite increasing evidence on the safety and clinical efficacy of the ESG, some limitations need to be addressed. Firstly, among the included studies there is a significant heterogeneity in study designs, patient population, and used ESG technique. This is one of the factors on which we did not conduct a meta-analysis. Secondly, the majority of the studies were conducted in a retrospective fashion. Thirdly, all the above-mentioned aspects limit the generalizability of our findings.

## 6. Future Research

Future research should focus on showing the long-term effects of ESG, but also the effects on the resolution of comorbidities. Secondly, we have to take into account that ESG can also be used as a revisional procedure after bariatric surgery. Further research should focus on comparing different treatment modalities and/or revisional surgical procedures with ESG.

## 7. Conclusions

In conclusion, endoscopic sleeve gastroplasty (ESG) is an effective and minimally invasive procedure for weight loss and the improvement of obesity-related comorbidities. It offers significant weight loss, with patients typically achieving 20–25% total body weight loss and 40–60% excess weight loss within the first year. Additionally, ESG leads to improvements in conditions like type 2 diabetes, hypertension, and dyslipidemia. With its promising outcomes, ESG presents a valuable alternative to traditional bariatric surgeries. However, future research should further determine the place of ESG in the treatment armamentarium of obesity specialists and bariatric and metabolic surgeons.

## Figures and Tables

**Table 1:** Overview of included studies.

Author (Year)	Study Design	Participants (n)	BMI (Mean, SD, Range)	Weight Loss (Mean, SD, Range)	Follow Up Period	Results (Mean, SD, Range)
De Moura et al. (2020) [4]	Multicenter prospective study	233	N/A	19.7% TBWL	12 months	Sustained weight loss, improved comorbidities
Matteo et al. (2024) [5]	Retrospective cohort	315	Median BMI: 36.1 (34.2, 39.4) females; 39.2 (36.0, 43.7) males	17–20% TBWL at 2 years, 16% at 5 years	24 months	Median %TBWL: 12.8% (6.41–19.4) at 24 months
Ryuet al. (2016) [20]	Multicenter retrospective study	34	Mean BMI: 34.8 ± 4.4	%TWL: 13.2 ± 3.9 at 6 months, 18.3 ± 5.5 at 1 year	12 months	%EWL: 51.9 ± 19.1 (6 months), 69.9 ± 29.9 (1 year)
Wang et al. (2020) [16]	Multicenter retrospective study	34	Mean BMI: 34.8 ± 4.8	%TWL: 13.2% ± 3.9 at 6 months, 18.3% ± 5.5 at 1 year	12 months	%EWL: 51.9% ± 19.1 (6 months), 69.9% ± 29.9 (1 year)
Lopez et al. (2017) [11]	Multicenter randomized trial	146	BMI: class III obesity	20.5% TBWL at 12 months	12 months	%EWL: ~20.5%
Abu Dayyeh BK et al. (2017) [8]	Case series	25	Mean BMI Mean 35.5 ± 2.6 kg/m^2^	EWL (mean): 53% ± 17% (6 months), 56% ± 23% (9 months), 54% ± 40% (12 months), 45% ± 41% (20 months)	9 months (range 5–20 months)	Sustained weight loss with a median of 45% EWL at 20 months, improved satiation, delayed gastric emptying, and increased insulin sensitivity
Graus Morales et al. (2018) [17]	Retrospective single-center study	148 (72 monitored 18 months)	35.11 ± 5.5 kg/m^2^ (all)	17.62 ± 9.22 kg (12 months, 17.53% WL)	12–18 months	79.25 ± 43% EWL (18 months)
De Moura et al. (2019) [4]	Retrospective review	N/A	N/A	Up to 20.9% total body weight loss	Up to 2 years	Favorable outcomes with 60.4% excess weight loss
Alqahtani et al. (2019) [21]	Prospective cohort study	1000	33.3 ± 4.5 kg/m^2^	6 months: 13.7% ± 6.8% TBWL 12 months: 15.0% ± 7.7% TBWL 18 months: 14.8% ± 8.5% TBWL	18 months	Significant weight loss within the first 18 months 13 of 17 diabetes cases in complete remission All 28 hypertension cases in remission
Alqahtani, A. et al. (2019) [22]	Prospective observational study	109	33 (mean)	6 months: 14.4 12 months: 16.2% ± 8.3% total weight loss 24 months: 13.7% ± 8.0% total weight loss	2 years	Weight loss of approximately 12 kg by 18 months
Asokkumar, R. et al. (2021) [23]	Case series	35	34 ± 4.9 kg/m^2^ (range not explicitly stated)	3 months: 14.5% ± 4.8% total body weight loss (TBWL) 6 months: 16.2% ± 4.9% TBWL	6 months	Weight loss at 3 months: 13.2 ± 4.8 kg Weight loss at 6 months: 14.1 ± 5.9 kg BMI reduction at 6 months: 5.7 ± 1.5 kg/m^2^
Badurdeen et al. (2021) [24]	Retrospective study	52	35.7 (mean) ± 2.02 kg/m^2^	ESG—only group: 7 months: 20.95 ± 3.21 kg lost, 20.51% ± 1.68% TBWL	12 months	Body fat reduction at 12 months: ESG-only: 10.54% ± 1.88%
Barrichello et al. (2019) [25]	Case series	193	34.11 ± 2.97 kg/m^2^	6 months: 14.25% ± 5.26% total weight loss (TWL) 1 year: 15.06% ± 5.22% TWL	6 months and a year	BMI reduction: initial: 34.11 ± 2.97 kg/m^2^ 6 months: 29.21 ± 2.64 kg/m^2^ 1 year: 28.91 ± 2.99 kg/m^2^ excess weight loss (EWL): 6 months: 56.15% ± 22.93% 1 year: 59.41% ± 25.69%
Carr et al. (2022) [26]	Cohort study	61	34.11 ± 2.97 kg/m^2^	BMI reduction: initial: 34.11 ± 2.97 kg/m^2^, 6 months: 29.21 ± 2.64 kg/m^2^	1 year	EWL: 6 months: 56.15% ± 22.93%; 1 year: 59.41% ± 25.69%
Cheskin et al. (2020) [27]	Prospective study	386	ESG: 37.8 (SD: 4.8) kg/m^2^ HIDLT: 37.3 (SD: 4.8) kg/m^2^	ESG: %TWL: 13.6% (SD: 7.6%) at 12 m HIDLT: %TWL: 0.8% (SD: 5.0%)	12 months	ESG resulted in significantly greater weight loss compared to HIDLT; no serious adverse events reported
Espinet-Coll, E. et al. (2022) [28]	Prospective study	38	Mean: 39.5 kg/m^2^	%TWL: 14.9% (SD: 6.8%) at 12 m	12 months	Suture patterns did not significantly affect weight loss outcomes
Espinet-Coll et al. (2020) [29]	Prospective study	88	Mean: 40.1(SD: 5.8) kg/m^2^	%TWL: 17.1% (SD: 7.2%) at 12 m	12 months	Sustained gastric volume reduction observed at 12 m post-ESG; no major adverse events reported
Farha et al. (2020) [30]	Retrospective	247	Mean: 38.2 kg/m^2^	%TWL: 16.4% at 12 m	12 months	Suturing the gastric fundus does not provide additional weight loss benefit
Fayad et al. (2019) [31]	Retrospective	137	ESG: 37.8 (SD: 4.8) kg/m^2^	ESG: 37.8 (SD: 4.8) kg/m^2^	12 months	ESG had fewer adverse events and shorter hospital stay
Fayad, L. et al. (2019) [32]	Retrospective	105	41.5 kg/m^2^, SD 8.2	9.9% (SD 2.4)	12 months	12 months
Fiorillo, C. et al. (2020) [33] Glaysher, M. A. et al. (2019) [34]	Retrospective Retrospective	4632	ESG: 35.7 (SD: 5.1) kg/m^2^ median 36.5 kg/m^2^ (range: 29.8–42.9)	ESG: %TWL: 15.1% (SD: 5.7%) at 6 m (range: 4.0–10.7%)	6 months 6 months	ESG resulted in greater weight loss BMI reduction was also greater (*p* = 0.019)
Gudur et al. (2022) [35]	Retrospective study	36,323	BMI: 39.1 ± 5.0 kg/m^2^	ESG: %TWL: 14.8% (SD: 4.9%) at 12 m;	12 months	ESG had better safety profile
Hajifathalian et al. (2021) [36]	Prospective	118	Mean: 40.0 kg/m^2^	%TWL: 16.3% at 12 m	12 months	Significant improvement in insulin resistance and hepatic steatosis after ESG
Hill et al. (2017) [37]	Prospective	21	Mean BMI: 37.6 kg/m	%TWL: 16.2% at 12 m	12 months	ESG proficiency improved significantly after 20 procedures
Jagtap, N. et al. (2021) [38]	Prospective	26	Mean: 39.1 kg/m^2^	TWL: 17.4% at 12 m	12 months	ESG found to be an effective treatment for obesity and non-alcoholic fatty liver disease
Brunaldi et al. (2022) [39]	Retrospective	100	BMI: 37.2 ± 5.3 kg/m^2^	TWL: 13.5% at 12 m	12 months	ESG is feasible in a non-academic community setting
Kumar, N. et al. (2018) [40]	Prospective	122	Mean BMI 37.4 ± 1.9 kg/m^2^	13.1 ± 1.3 kg	12 months	ESG showed significant weight loss over 12 months
Li, R. et al. (2021) [41]	Prospective	21	49.9 ± 14.4 kg/m^2^	Weight loss: 17.5 ± 14.6 kg	12 months	ESG was successfully performed in all patients without intraoperative complications
Lopez-Nava, G. et al. (2022) [42]	Retrospective	435	Mean 45.8 kg/m^2^	20.5%	12 months	ESG was effective in all three obesity classes, with higher weight loss in class III patients
Lopez-Nava, G. et al. (2017) [43]	Prospective	248	37.8 ± 5.6 kg/m^2^	TBWL: 18.6% (95% CI: 15.7–21.5)	24 months	ESG effectively induced weight loss up to 24 months in moderately obese patients
Neto, M. G. et al. (2019) [44]	Prospective	233	34.7 ± 2.6 kg/m^2^	19.7% (±5.7)	12 months	ESG resulted in significant short-term weight loss, with an average %TBWL of 19.7% at 12 months
Neto, M. G. et al. (2020) [45]	Prospective	1828	30–40 kg/m^2^	18.2%	12 months	ESG resulted in significant weight loss and had a low complication rate
Pizzicannella, M. et al. (2019) [46]	Prospective	133	43.2 ± 8.6 kg/m^2^	%EWL: 19.3 ± 13.4, %TWL: 8.9 ± 6.1	12 months	Weight loss correlated with ESG durability: Patients with intact ESG had the highest %EWL and %TWL at 6 and 12 months
Sarkar, A. et al. (2022) [47]	Retrospective	91	38.7 kg/m^2^ (range: 31.2–57.6)	17.4%	12 months	ESG in new bariatric centers yielded comparable weight loss and metabolic outcomes to experienced centers
Sartoretto, A. et al. (2018) [48]	Retrospective	112	37.9 ± 6.7 kg/m^2^	%TBWL: 14.9 ± 6.1	6 months	Male patients and those with higher baseline BMI tended to lose more weight
Saumoy, M., y col (2017) [49]	Prospective	128	38.92 ± 6.95 kg/m^2^ (range: 30.02–68.04)	%TBWL: 15.80 ± 9.50	12 months	ESG can be efficiently and safely mastered after approximately 55 cases
Sharaiha, R. Z. et al. (2020) [50]	Retrospective	216	39 ± 6 kg/m^2^	%TBWL: 15.9% (95% CI: 11.7–20.5, *p* < 0.001)	5 years	90% of patients maintained at least 5% TBWL at 5 years, meeting ASGE and ASMBS criteria for primary bariatric intervention

Abbreviations: BMI = body mass index, EWL = excess weight loss, TWL = Total Weight Loss, TBWL = total body weight loss, LGP = Laparoscopic Greater Curvature Plication, LSG = laparoscopic sleeve gastrectomy, RYGB = Roux-en-Y Gastric Bypass, LAGB = Laparoscopic Adjustable Gastric Banding, ESG = endoscopic sleeve gastroplasty.

## Data Availability

No new data were created or analyzed in this study.

## References

[1] World Health Organization Obesity. https://www.who.int/health-topics/obesity#tab=tab_1.

[2] Ng M., Fleming T., Robinson M. (2014). Global, regional, and national prevalence of overweight and obesity in children and adults. Lancet.

[3] Heymsfield S.B., Wadden T. (2017). Mechanisms, Pathophysiology, and Management of Obesity. N. Engl. J. Med..

[4] de Moura D.T.H., Barrichello S., de Moura E.G.H., de Souza T.F., Neto M.D.P.G., Grecco E., Sander B., Hoff A.C., Matz F., Ramos F. (2020). Endoscopic sleeve gastroplasty in the management of weight regain after sleeve gastrectomy. Endoscopy.

[5] Matteo M.V., Bove V., Ciasca G., Carlino G., Di Santo R., Vinti L., Boškoski I. (2024). Success Predictors of Endoscopic Sleeve Gastroplasty. Obes. Surg..

[6] Yanovski S.Z., Yanovski J.A. (2014). Long-term drug treatment for obesity: A systematic and clinical review. JAMA.

[7] Mauro A., Lusetti F., Scalvini D., Bardone M., De Grazia F., Mazza S., Pozzi L., Ravetta V., Rovedatti L., Sgarlata C. (2023). A Comprehensive Review on Bariatric Endoscopy: Where We Are Now and Where We Are Going. Medicina.

[8] Abu Dayyeh B.K., Acosta A., Camilleri M., Mundi M.S., Rajan E., Topazian M.D., Gostout C.J. (2017). Endoscopic Sleeve Gastroplasty Alters Gastric Physiology and Induces Loss of Body Weight in Obese Individuals. Clin. Gastroenterol. Hepatol..

[9] Galvão-Neto M.D., Grecco E., Souza T.F., Quadros L.G., Silva L.B., Campos J.M. (2016). ENDOSCOPIC SLEEVE GASTROPLASTY-MINIMALLY INVASIVE THERAPY FOR PRIMARY OBESITY TREATMENT. Arq. Bras. Cir. Dig..

[10] ASGE/ASMBS Task Force on Endoscopic Bariatric Therapy (2011). A pathway to endoscopic bariatric therapies. Surg. Obes. Relat. Dis..

[11] Lopez-Nava G., Galvão M.P., Bautista-Castaño I., Fernandez-Corbelle J.P., Trell M., Lopez N. (2017). Endoscopic Sleeve Gastroplasty for Obesity Treatment: Two Years of Experience. Arq. Bras. Cir. Dig..

[12] Mohan B.P., Asokkumar R., Khan S.R., Kotagiri R., Sridharan G.K., Chandan S., Adler D.G. (2020). Outcomes of endoscopic sleeve gastroplasty, how does it compare to laparoscopic sleeve gastrectomy? A systematic review and meta-analysis. Endosc. Int. Open.

[13] Hedjoudje A., Dayyeh B.K.A., Cheskin L.J., Adam A., Neto M.G., Badurdeen D., Kumbhari V. (2020). Efficacy and Safety of Endoscopic Sleeve Gastroplasty, A Systematic Review and Meta-Analysis. Clin. Gastroenterol. Hepatol..

[14] Fogel R., De Fogel J., Bonilla Y., De La Fuente R. (2008). Clinical experience of transoral suturing for an endoluminal vertical gastroplasty: 1-year follow-up in 64 patients. Gastrointest. Endosc..

[15] Dials J., Demirel D., Halic T., De S., Ryason A., Kundumadam S., Al-Haddad M., Gromski M.A. (2022). Hierarchical task analysis of endoscopic sleeve gastroplasty. Surg. Endosc..

[16] Wang J.-W., Chen C.-Y. (2020). Current status of endoscopic sleeve gastroplasty: An opinion review. World J. Gastroenterol..

[17] Morales J.G., Pérez L.C., Marques A., Arribas B.M., Arribas R.B., Ramo E., Escalada C., Arribas C., Himpens J. (2018). Modified endoscopic gastroplasty for the treatment of obesity. Surg. Endosc..

[18] Maselli D.B., Hoff A.C., Kucera A., Weaver E., Sebring L., Gooch L., Walton K., Lee D., Cratty T., Beal S. (2023). Endoscopic sleeve gastroplasty in class III obesity: Efficacy, safety, and durability outcomes in 404 consecutive patients. World J. Gastrointest. Endosc..

[19] Dayyeh B.K.A., Bazerbachi F., Vargas E.J., Sharaiha R.Z., Thompson C.C., Thaemert B.C., Wilson E.B. (2022). Endoscopic sleeve gastroplasty for treatment of class 1 and 2 obesity (MERIT): A prospective, multicentre, randomised trial. Lancet.

[20] Ryu S.J., Kim B.W., Kim B.G., Kim J.H., Kim J.S., Kim J.I., Kim W. (2016). Endoscopic submucosal dissection versus surgical resection for early gastric cancer: A retrospective multicenter study on immediate and long-term outcome over 5 years. Surg. Endosc..

[21] Alqahtani A., Elahmedi M., Alqahtani Y.A., Al-Darwish A. (2019). Endoscopic sleeve gastroplasty in 109 consecutive children and adolescents with obesity: Two-year outcomes of a new modality. Am. J. Gastroenterol..

[22] Alqahtani A., Al-Darwish A., Mahmoud A.E., Alqahtani Y.A., Elahmedi M. (2019). Short-term outcomes of endoscopic sleeve gastroplasty in 1000 consecutive patients. Gastrointest. Endosc..

[23] Asokkumar R., Lim C.H., Tan A.S., Lee P.C., Eng A., Tan J., Lopez-Nava G., Ganguly S., Chang J., Khor C. (2021). Safety and early efficacy of endoscopic sleeve gastroplasty (ESG) for obesity in a multi-ethnic Asian population in Singapore. JGH Open.

[24] Badurdeen D., Hoff A.C., Hedjoudje A., Adam A., Itani M.I., Farha J., Abbarh S., Kalloo A.N., Khashab M.A., Singh V.K. (2021). Endoscopic sleeve gastroplasty plus liraglutide versus endoscopic sleeve gastroplasty alone for weight loss. Gastrointest. Endosc..

[25] Barrichello S., de Moura D.T.H., de Moura E.G.H., Jirapinyo P., Hoff A.C., Fittipaldi-Fernandez R.J., Thompson C.C. (2019). Endoscopic sleeve gastroplasty in the management of overweight and obesity: An international multicenter study. Gastrointest. Endosc..

[26] Carr P., Keighley T., Petocz P., Blumfield M., Rich G.G., Cohen F., Soni A., Maimone I.R., Fayet-Moore F., Isenring E. (2022). Efficacy and safety of endoscopic sleeve gastroplasty and laparoscopic sleeve gastrectomy with 12+ months of adjuvant multidisciplinary support. BMC Prim. Care.

[27] Cheskin L.J., Hill C., Adam A., Fayad L., Dunlap M., Badurdeen D., Koller K., Bunyard L., Frutchey R., Al-Grain H. (2020). Endoscopic sleeve gastroplasty versus high-intensity diet and lifestyle therapy: A case-matched study. Gastrointest. Endosc..

[28] Espinet-Coll E., Díaz-Galán P., Nebreda-Durán J., Gómez-Valero J.A., Vila-Lolo C., Bautista-Altamirano C., Bargalló-García A., Galvao-Neto M., Muñoz-Navas M., Bargalló-Carulla D. (2022). Persistence of sutures and gastric reduction after endoscopic sleeve gastroplasty: Radiological and endoscopic assessment. Obes. Surg..

[29] Espinet-Coll E., Nebreda-Durán J., Galvao-Neto M., Bautista-Altamirano C., Diaz-Galán P., Gómez-Valero J.A., Vila-Lolo C., Guirola-Puche M.A., Fernández-Huélamo A., Bargalló-Carulla D. (2020). Suture pattern does not influence outcomes of endoscopic sleeve gastroplasty in obese patients. Endosc. Int. Open.

[30] Farha J., McGowan C., Hedjoudje A., Itani M.I., Abbarh S., Simsek C., Ichkhanian Y., Vulpis T., James T.W., Fayad L. (2020). Endoscopic sleeve gastroplasty: Suturing the gastric fundus does not confer benefit. Endoscopy.

[31] Fayad L., Adam A., Schweitzer M., Cheskin L.J., Ajayi T., Dunlap M., Badurdeen D.S., Hill C., Paranji N., Lalezari S. (2019). Endoscopic sleeve gastroplasty versus laparoscopic sleeve gastrectomy: A case-matched study. Gastrointest. Endosc..

[32] Fayad L., Cheskin L.J., Adam A., Badurdeen D.S., Hill C., Agnihotri A., Dunlap M., Simsek C., Khashab M.A., Kalloo A.N. (2019). Endoscopic sleeve gastroplasty versus intragastric balloon insertion: Efficacy, durability, and safety. Endoscopy.

[33] Fiorillo C., Quero G., Vix M., Guerriero L., Pizzicannella M., Lapergola A., D’uRso A., Swanstrom L., Mutter D., Dallemagne B. (2020). 6-month gastrointestinal quality of life (QoL) results after endoscopic sleeve gastroplasty and laparoscopic sleeve gastrectomy: A propensity score analysis. Obes. Surg..

[34] Glaysher M.A., Moekotte A.L., Kelly J. (2019). Endoscopic sleeve gastroplasty: A modified technique with greater curvature compression sutures. Endosc. Int. Open.

[35] Gudur A.R., Geng C.X., Kshatri S., Martin D., Haug R., Radlinski M., Lei Y., Buerlein R.C., Strand D.S., Sauer B.G. (2022). Comparison of endoscopic sleeve gastroplasty versus surgical sleeve gastrectomy: A metabolic and bariatric surgery accreditation and quality improvement program database analysis. Gastrointest. Endosc..

[36] Hajifathalian K., Mehta A., Ang B., Skaf D., Shah S.L., Saumoy M., Dawod Q., Dawod E., Shukla A., Aronne L. (2021). Improvement in insulin resistance and estimated hepatic steatosis and fibrosis after endoscopic sleeve gastroplasty. Gastrointest. Endosc..

[37] Hill C., El Zein M., Agnihotri A., Dunlap M., Chang A., Agrawal A., Kumbhari V. (2017). Endoscopic sleeve gastroplasty: The learning curve. Endosc. Int. Open.

[38] Jagtap N., Kalapala R., Katakwar A., Sharma M., Aslam M., Gupta R., Rao P.N., Goud R., Tandan M., Kanakagiri H. (2021). Endoscopic sleeve gastroplasty—Minimally invasive treatment for non-alcoholic fatty liver disease and obesity. Indian J. Gastroenterol..

[39] Brunaldi V.O., Neto M.G. (2022). Endoscopic sleeve gastroplasty: A narrative review on historical evolution, physiology, outcomes, and future standpoints. Chin. Med. J..

[40] Kumar N., Abu Dayyeh B.K., Breviere G.L.-N., Neto M.P.G., Sahdala N.P., Shaikh S.N., Hawes R.H., Gostout C.J., Goenka M.K., Orillac J.R. (2017). Endoscopic sutured gastroplasty: Procedure evolution from first-in-man cases through current technique. Surg. Endosc..

[41] Li R., Veltzke-Schlieker W., Adler A., Specht M., Eskander W., Ismail M., Badakhshi H., Galvao M.P., Zorron R. (2021). Endoscopic sleeve gastroplasty (ESG) for high-risk patients, high body mass index (>50 kg/m^2^) patients, and contraindication to abdominal surgery. Obes. Surg..

[42] Lopez-Nava G., Laster J., Negi A., Fook-Chong S., Bautista-Castaño I., Asokkumar R. (2021). Endoscopic sleeve gastroplasty (ESG) for morbid obesity: How effective is it?. Surg. Endosc..

[43] Lopez-Nava G., Sharaiha R.Z., Vargas E.J., Bazerbachi F., Manoel G.N., Bautista-Castaño I., Acosta A., Topazian M.D., Mundi M.S., Kumta N. (2017). Endoscopic sleeve gastroplasty for obesity: A multicenter study of 248 patients with 24 months follow-up. Obes. Surg..

[44] Neto M.G., Silva L.B., de Quadros L.G., Grecco E., Filho A.C., de Amorim A.M.B., de Santana M.F., dos Santos N.T., de Lima J.H.F., de Souza T.F. (2020). Brazilian consensus on endoscopic sleeve gastroplasty. Obes. Surg..

[45] Neto M.G., Moon R.C., de Quadros L.G., Grecco E., Filho A.C., de Souza T.F., Mattar L.A., de Sousa J.A.G., Abu Dayyeh B.K., Morais H. (2020). Safety and short-term effectiveness of endoscopic sleeve gastroplasty using overstitch: Preliminary report from a multicenter study. Surg. Endosc..

[46] Pizzicannella M., Lapergola A., Fiorillo C., Spota A., Mascagni P., Vix M., Mutter D., Costamagna G., Marescaux J., Swanström L. (2020). Does endoscopic sleeve gastroplasty stand the test of time? Objective ESG appearance and weight loss in a large group of patients. Surg. Endosc..

[47] Sarkar A., Tawadros A., Andalib I., Shahid H.M., Tyberg A., Alkhiari R., Gaidhane M., Kedia P., John E.S., Bushe B. (2022). Safety and efficacy of endoscopic sleeve gastroplasty for obesity management in new bariatric endoscopy programs: A multicenter international study. Ther. Adv. Gastrointest. Endosc..

[48] Sartoretto A., Sui Z., Hill C., Dunlap M., Rivera A.R., Khashab M.A., Kalloo A.N., Fayad L., Cheskin L.J., Marinos G. (2018). Endoscopic sleeve gastroplasty is a reproducible and effective endoscopic bariatric therapy suitable for widespread clinical adoption: A large, international multicenter study. Obes. Surg..

[49] Saumoy M., Schneider Y., Zhou X.K., Shukla A., Kahaleh M., Aronne L., Sharaiha R.Z. (2018). A single-operator learning curve analysis for the endoscopic sleeve gastroplasty. Gastrointest. Endosc..

[50] Sharaiha R.Z., Hajifathalian K., Kumar R., Saunders K., Mehta A., Ang B., Skaf D., Shah S., Herr A., Igel L. (2021). Five-year outcomes of endoscopic sleeve gastroplasty for the treatment of obesity. Clin. Gastroenterol. Hepatol..

[51] PRISMA PRISMA: Transparent Reporting of Systematic Reviews and Meta-Analyses [Internet]. https://www.prisma-statement.org/.

[52] Vargas E.J., Rizk M., Gomez-Villa J., Edwards P.K., Jaruvongvanich V., Storm A.C., Acosta A., Lake D., Fidler J., Bharucha A.E. (2022). Effect of endoscopic sleeve gastroplasty on gastric emptying, motility and hormones: A comparative prospective study. Gut.

[53] James T.W., Reddy S., Vulpis T., McGowan C.E. (2019). Endoscopic Sleeve Gastroplasty Is Feasible, Safe, and Effective in a Non-academic Setting: Short-Term Outcomes from a Community Gastroenterology Practice. Obes. Surg..

[54] Sullivan S., Edmundowicz S. (2017). Early experience with endoscopic sleeve gastroplasty and hints at mechanisms of action. Clin. Gastroenterol. Hepatol..

[55] Ward Z.J., Bleich S.N., Cradock A.L., Barrett J.L., Giles C.M., Flax C., Long M.W., Gortmaker S.L. (2019). Projected U.S. State-Level Prevalence of Adult Obesity and Severe Obesity. N. Engl. J. Med..

[56] Sharaiha R.Z., Kumta N.A., Saumoy M., Desai A.P., Sarkisian A.M., Benevenuto A., Tyberg A., Kumar R., Igel L., Verna E.C. (2017). Endoscopic Sleeve Gastroplasty Significantly Reduces Body Mass Index and Metabolic Complications in Obese Patients. Clin. Gastroenterol. Hepatol..

[57] Sharaiha R.Z., Campos J.M., Fayad L., Familiar P., Kumbhari V., Novikov A.A., Besharati S., Ghandour B., Maselli D.B., Vargas E.J. (2023). Revisional endoscopic gastroplasty (re-ESG) after weight recurrence post-endoscopic sleeve gastroplasty or laparoscopic sleeve gastrectomy: A systematic review. Endosc. Int. Open.

[58] Abu Dayyeh B.K., Stier C., Alqahtani A., Sharaiha R., Bandhari M., Perretta S., Jirapinyo S.P., Prager G., Cohen R.V. (2024). IFSO Bariatric Endoscopy Committee evidence-based review and position statement on endoscopic sleeve gastroplasty for obesity management. Obes. Surg..

